# Factor XI Inhibition for Ischemic Stroke Secondary Prevention: Pharmacology, Clinical Evidence, and Future Directions

**DOI:** 10.1002/jcph.70266

**Published:** 2026-08-14

**Authors:** Federica Ferrari, Roberto Federico Villa

**Affiliations:** ^1^ Department of Health Sciences School of Medicine Univ ersità del Piemonte Orientale “Amedeo Avogadro” Novara Italy; ^2^ Department of Biology and Biotechnology Università di Pavia Pavia Italy

**Keywords:** antithrombotic therapy, FXIa inhibitors, hemostasis‐sparing anticoagulation, ischemic stroke, secondary prevention

## Abstract

Ischemic stroke remains a leading cause of death and disability worldwide, and bleeding (particularly intracranial hemorrhage) continues to limit conventional antithrombotic therapy in secondary prevention, even with direct oral anticoagulants. Factor XI (FXI) has emerged as a promising target: it amplifies thrombin generation and stabilizes pathological thrombi while contributing only marginally to physiological hemostasis, as illustrated by the mild bleeding phenotype of congenital FXI deficiency. This biological dissociation underpins the concept of hemostasis‐sparing anticoagulation. Three pharmacological classes are being studied, with distinct pharmacokinetic, pharmacodynamic, and drug‐drug interaction profiles: small‐molecule oral FXIa inhibitors (asundexian and milvexian), FXI antisense oligonucleotides (fesomersen), and monoclonal antibodies targeting FXI/FXIa (abelacimab and osocimab). Oral small molecules offer rapid, reversible target engagement particularly suited to chronic cerebrovascular prevention. While phase II trials (PACIFIC‐Stroke, AXIOMATIC‐SSP) showed neutral primary endpoints with reassuring safety, the phase III OCEANIC‐STROKE trial demonstrated that asundexian 50 mg once daily, added to antiplatelet therapy, reduced recurrent ischemic stroke by 26% (HR 0.74; 95% CI 0.65–0.84) without significant bleeding excess in non‐cardioembolic stroke; the ongoing LIBREXIA‐STROKE is testing milvexian 25 mg twice daily in a similar setting. Conversely, OCEANIC‐AF showed that FXIa inhibition alone is insufficient versus apixaban in atrial fibrillation, indicating dependence upon clinical setting and suggesting the need for a precision‐medicine approach integrating clinical, imaging, and biomarker‐based patient stratification. This narrative review integrates pathophysiological rationale, comparative pharmacology of all FXI‐targeting classes, trial evidence, and patient stratification, providing an up‐to‐date reference for FXI/FXIa inhibition in secondary ischemic stroke prevention.

## Introduction

Ischemic stroke is a leading cause of death and long‐term disability worldwide. The Global Burden of Disease (GBD) Study 2021 reported ∼11.9 million incident events, 7.3 million deaths, and 160.5 million disability‐adjusted life years (DALYs) globally, ranking stroke as the third cause of death and the fourth of DALYs.^1^ Ischemic stroke represents ∼65% of all strokes, with projections to further increase through 2035, particularly in low‐ and middle‐income countries, which account for >85% of stroke‐related deaths and DALYs.^1^

Survivors face a recurrence risk of ∼8% in the first year and of 2%–4% annually thereafter, that is, four‐fold higher than in stroke‐free individuals.^2^ Five‐year cumulative recurrence ranges from 20% to 25%, particularly in large‐artery atherosclerosis and cardioembolic mechanisms.^3^ Recurrent stroke increases disability, mortality, dementia, and cognitive decline.^4^

Secondary prevention relies on mechanism‐tailored antithrombotic therapy but remains constrained by bleeding risk, particularly intracranial hemorrhage in elderly patients with cerebral small‐vessel disease, microbleeds, or prior hemorrhagic transformation.^5^, ^6^ Moreover, 25%–30% of ischemic strokes remain cryptogenic, and empirical anticoagulation has not proven superior to antiplatelet therapy in Embolic Strokes with Undetermined Source (ESUS).^7^, ^8^

In this context, factor XI (FXI) inhibition offers a potential solution: FXI amplifies thrombin generation and stabilizes thrombi contributing marginally to physiological hemostasis, as shown by the mild bleeding phenotype of congenital FXI deficiency. This “hemostasis‐sparing” profile may attenuate pathological thrombosis while preserving tissue‐factor‐driven hemostasis.

FXI inhibition has already been the subject of authoritative reviews, most comprehensively by Capodanno et al across the spectrum of venous and arterial thromboembolism^9^ and within neurology by Nolte.^10^ These contributions, however, address FXI inhibition primarily as a cross‐indication cardiovascular strategy and were written before phase III evidence in cerebrovascular secondary prevention became available. The present review differs in three respects. First, it is centered specifically on secondary prevention after ischemic stroke, a setting in which the comparator is placebo on an antiplatelet background rather than a direct oral anticoagulant (DOAC), so that the benefit‐risk equation differs substantially from that of atrial fibrillation or venous thromboembolism. Second, it incorporates the most recent evidence, including the phase III OCEANIC‐STROKE results, the futility‐based discontinuation of LIBREXIA‐ACS, and the meta‐analyses published to date. Third, it goes beyond a descriptive account of the drug class to a critical appraisal of how the pharmacodynamic and pharmacokinetic properties of the different FXI‐targeting classes condition their applicability in stroke patients, and proposes stratification criteria to identify the patients most likely to derive net benefit.

This narrative review, based on PubMed literature up to April 2026, not only examines the rationale, pharmacology, and clinical evidence for FXI inhibitors in ischemic stroke prevention, but is also intended to critically analyze the intrinsic potential characteristics of the underlying pharmacodynamic mechanisms within the ischemic stroke settings, discussing stratification strategies to optimize the benefit‐risk profile.

## Pathophysiological Basis and Therapeutic Rationale for Factor XI Inhibition

### The Coagulation Cascade and Currently Available Antithrombotic Agents

**Figure 1:**
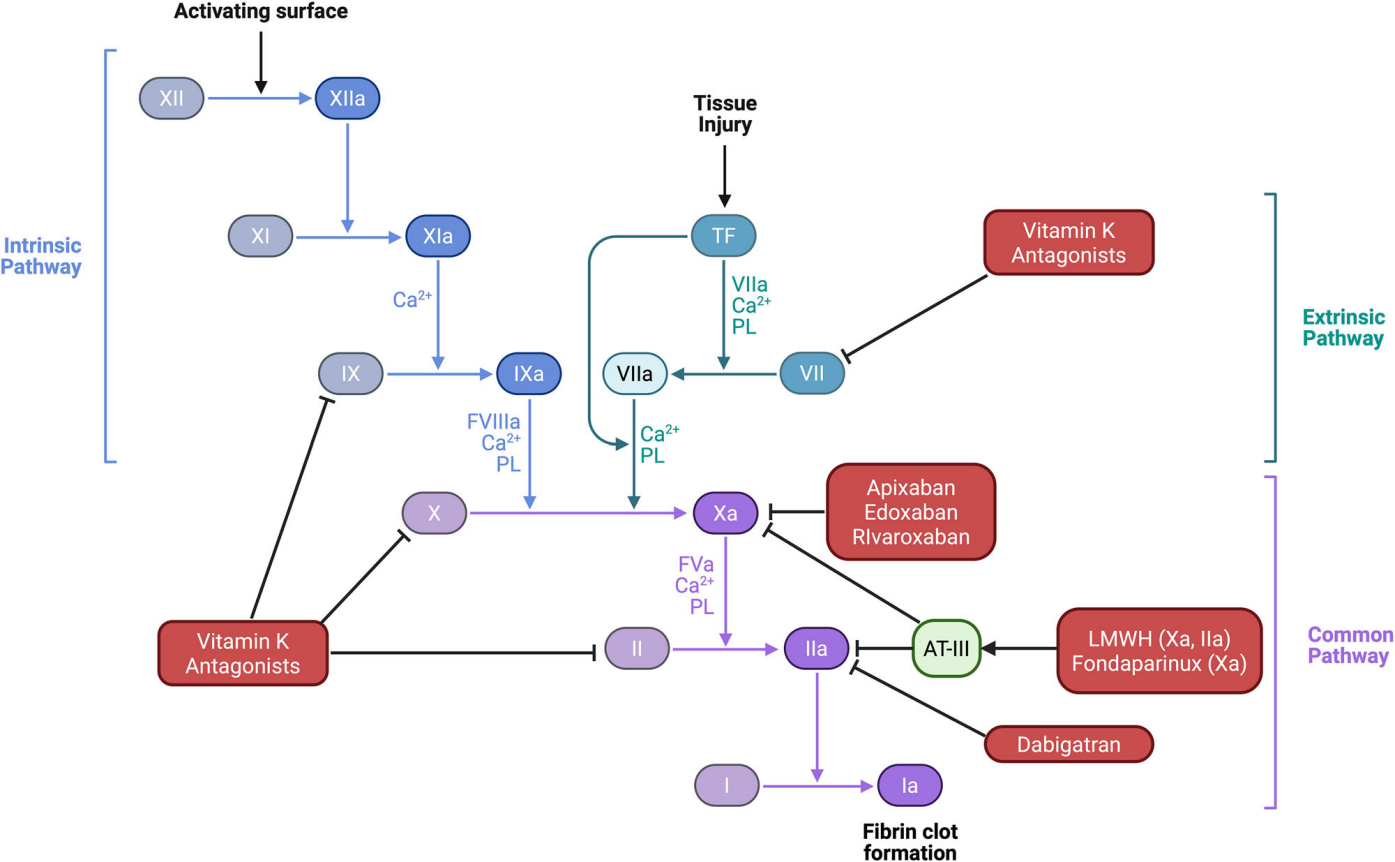
Schematic representation of the classic coagulation cascade and sites of anticoagulant action. The cascade is divided into the intrinsic (contact activation), extrinsic (tissue factor), and common pathways. The intrinsic pathway is initiated by factor XII (FXII) following contact with activating surfaces, while the extrinsic pathway is triggered by tissue factor (TF) following vascular injury. Both converge on the common pathway at the level of Factor X activation. The figure highlights the mechanism of action for several classes of clinical anticoagulants: Vitamin K antagonists inhibit the synthesis of Factors II, VII, IX, and X; direct oral anticoagulants (DOACs) specifically target either Factor Xa (apixaban, edoxaban, and rivaroxaban) or thrombin (dabigatran); and heparin‐based therapies (LMWH and fondaparinux) act via antithrombin‐III (AT‐III) to neutralize FXa and FIIa. Created with BioRender.com.

The coagulation system is central to both physiological hemostasis and pathological thrombus formation. In the traditional model, coagulation is initiated through two pathways: the extrinsic pathway, triggered by tissue factor (TF), and the intrinsic pathway, initiated by factor XII (FXII). These pathways converge on factor X (FX) activation, leading to thrombin generation, fibrin formation, and clot stabilization (Figure 1).

Current anticoagulants target key nodes of this cascade. Vitamin K antagonists (VKAs) such as warfarin inhibit synthesis of factors II, VII, IX, and X. Heparins potentiate antithrombin III and inhibit thrombin and/or active FX. Oral direct thrombin inhibitors such as dabigatran prevent fibrinogen‐to‐fibrin conversion. Direct oral FXa inhibitors (apixaban, edoxaban, and rivaroxaban) inhibit circulating FXa and reduce thrombin generation from prothrombin. Fondaparinux, a parenteral indirect FXa inhibitor, binds antithrombin with high affinity and enhances its neutralization of FXa.^11^

The introduction of DOACs improved the therapeutic possibilities through more predictable pharmacokinetics, fewer interactions, and lower intracranial bleeding rates than VKAs. DOACs are now first‐line therapy for stroke prevention in non‐valvular atrial fibrillation, showing superior or non‐inferior efficacy versus warfarin.^12^ In the meta‐analysis by Ruff et al^13^ of 71,683 patients from RE‐LY, ROCKET‐AF, ARISTOTLE, and ENGAGE AF‐TIMI 48 trials, DOACs reduced stroke or systemic embolism by 19% (RR 0.81; 95% CI 0.73–0.91; *P* <.0001), hemorrhagic stroke by 51% (RR 0.49; 95% CI 0.38–0.64), and all‐cause mortality by 10% (RR 0.90; 95% CI 0.85–0.95; *P* = .0003).

Despite these advances, current anticoagulants remain limited by bleeding. Even DOACs, while safer than VKAs for intracerebral hemorrhage (ICH), may cause clinically relevant and major bleeding. Apixaban, dabigatran 110 mg BID and edoxaban showed significantly lower major bleeding than warfarin, whereas dabigatran 150 mg BID and rivaroxaban did not.^12^ In particular, gastrointestinal bleeding remains an important concern, although the risk differs among DOACs: apixaban has the most favorable gastrointestinal safety profile, with lower bleeding than dabigatran (HR 0.81; 95% CI 0.70–0.94), edoxaban (HR 0.77; 95% CI 0.66–0.91), and rivaroxaban (HR 0.72; 95% CI 0.66–0.79).^14^ Apixaban is the only DOAC that does not significantly increase gastrointestinal bleeding versus warfarin, whereas edoxaban and dabigatran appear dose dependent.^15^

These concerns highlight important DOAC limitations for the pharmacological treatment in specific patient populations. In end‐stage renal disease (creatinine clearance <15 mL/min) or dialysis, evidence remains limited because pivotal trials excluded these patients.^15^ This pattern of exclusion is not unique to DOAC studies: across most contemporary cardiovascular and antithrombotic trials, patients with severe renal impairment are routinely excluded despite carrying the highest absolute thromboembolic and bleeding risk, so that the populations most in need of evidence remain the least represented in randomized data. DOACs are also not recommended in pregnancy or breastfeeding due to insufficient safety data.^16^ Safer anticoagulant strategies therefore remain an unmet need, particularly in stroke prevention, where ischemic benefit must be balanced against intracranial bleeding risk.

### Evolving Understanding of the Coagulation Cascade

Over the past two decades, the classical coagulation model has been revised in favor of a cell‐based model.^17^ In this framework, coagulation occurs dynamically on cellular surfaces, particularly on TF‐bearing cells and activated platelets, and is divided into three overlapping phases: initiation, amplification, and propagation.

During initiation, TF exposure at the injury site forms the TF‐FVIIa complex, activates FX, and generates a small amount of thrombin sufficient to initiate fibrin formation and activate platelets. In the amplification phase, thrombin activates FV and FVIII, and FXI on platelets, preparing the surfaces for efficient complex assembly. In the propagation phase, activated platelets support intense thrombin generation through the intrinsic pathway, yielding robust fibrin production and clot stabilization.^18^

**Figure 2:**
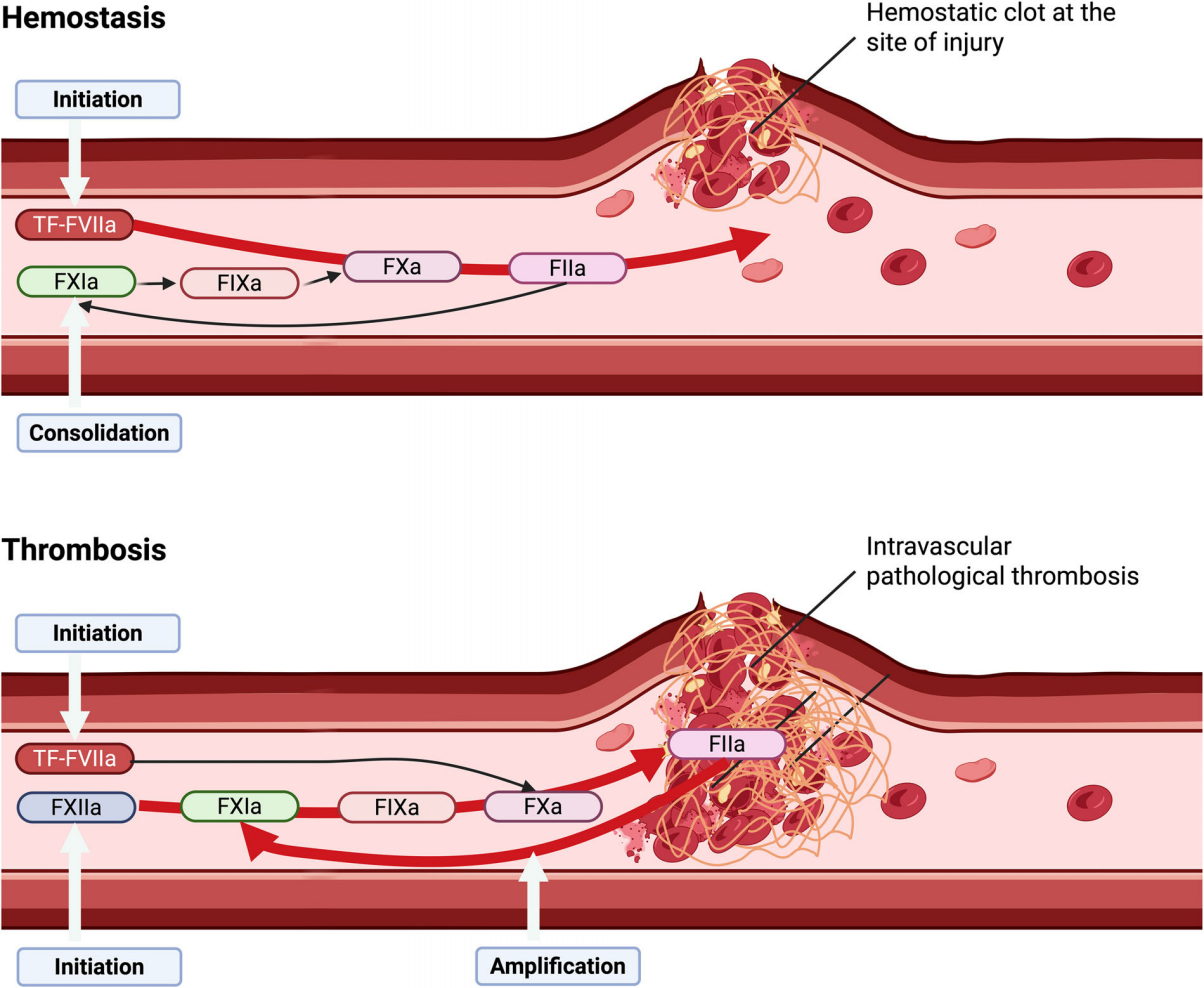
Differential roles of factor XI in physiological hemostasis and pathological thrombosis. The diagram illustrates two distinct pathways of the coagulation cascade. Bold red arrows indicate the dominant flux of thrombin generation and clot growth in each setting, whereas thin black arrows denote ancillary or feedback enzymatic reactions of comparatively minor quantitative contribution. (Top) In physiological hemostasis, the process is initiated by exposure of subendothelial tissue factor (TF) at the site of vascular injury, which forms the TF‐FVIIa complex (Initiation). This complex activates factor X, generating a small amount of thrombin (FIIa) sufficient to produce a localized fibrin‐platelet plug that seals the vessel injury. FXIa contributes only secondarily, through a thrombin‐mediated feedback loop that sustains additional FIX activation and reinforces clot stability (Consolidation), without being indispensable for primary hemostasis. (Bottom) Pathological intravascular thrombosis. Thrombus formation can be triggered either by intravascular TF exposure or by contact‐pathway activation of FXII to FXIIa (Initiation). Thrombin produced during the initiation phase activates FXI, igniting an amplification loop that sustains massive thrombin generation and extensive fibrin deposition (Amplification). The resulting intraluminal thrombus propagates along the vessel and may ultimately obstruct blood flow. The TF‐FVIIa contribution to thrombin generation in this setting is comparatively limited. Created with BioRender.com.

Although the same molecular machinery underlies both physiological hemostasis and pathological thrombosis, the relative contribution of each phase differs between the two settings (Figure 2). Hemostasis at sites of vascular injury is driven primarily by TF‐FVIIa‐initiated thrombin generation, with FXI playing only a minor, ancillary role in stabilizing—or “consolidating”—the hemostatic plug. Pathological intravascular thrombosis, by contrast, depends on the FXI/FXIa‐mediated amplification loop, in which thrombin feedback activates FXI to sustain and propagate thrombus growth on the surface of activated platelets.^19^, ^20^ This dichotomy provides the conceptual basis for decoupling thrombosis from hemostasis through selective inhibition of contact‐pathway components. Conventional anticoagulants inhibit thrombin or FXa, that is, factors essential to both normal hemostatic clot formation and pathological thrombosis, and therefore inevitably impair hemostasis at therapeutic doses. By contrast, inhibition of selected components of the contact pathway may attenuate thrombus growth while preserving the TF‐driven hemostatic response needed to prevent bleeding.

This conceptual shift is particularly relevant in cerebrovascular disease, where the major challenge is preventing pathological thromboembolism while minimizing ICH risk. A target predominantly involved in thrombus propagation rather than primary hemostatic initiation is therefore attractive.

### Factor XI: Structure, Function, and Rationale for Therapeutic Targeting

FXI is one of the most promising targets within this context. FXI is a 160‐kDa serine protease zymogen circulating as a disulfide‐linked homodimer composed of two identical subunits. Each subunit contains four N‐terminal apple domains and one C‐terminal catalytic domain mediating interactions with high‐molecular‐weight kininogen, thrombin, FIX, FXII, platelets, and heparin. Upon activation to FXIa, FXI cleaves FIX, amplifying thrombin generation through the intrinsic pathway^21^ (Figure 2).

FXI can be activated through at least two biological mechanisms. The first is activation by FXIIa within the intrinsic contact pathway, particularly relevant when blood is exposed to negatively charged or artificial surfaces. The second, and likely more significant in most thrombotic settings, is activation by thrombin itself, creating a positive feedback loop that enhances thrombin generation.^22^ This loop is facilitated by activated platelet surfaces and augmented by polyanions such as extracellular RNA, DNA, neutrophil extracellular traps, and platelet‐derived polyphosphates within the thrombotic and inflammatory microenvironment.^20^

Although FXI contributes to coagulation, its role in physiological hemostasis is limited and context‐dependent (Figure 2). In vivo, hemostatic clot initiation is driven primarily by the TF‐FVIIa complex, which can activate both FX and FIX and support sufficient thrombin generation even in the absence of normal FXI activity.^20^ This explains why congenital FXI deficiency (hemophilia C) has a milder and more variable bleeding phenotype than hemophilia A or B: spontaneous bleeding is uncommon, and severe bleeding into joints, muscles, the gastrointestinal tract, or the central nervous system is generally not observed. Bleeding occurs mainly after trauma or surgery, particularly in tissues with high fibrinolytic activity such as the oral cavity, nasopharynx, and genitourinary tract.^23^, ^24^

FXI deficiency thus provides a “natural experiment” supporting the view that FXI is more relevant to pathological thrombosis than to physiological hemostasis. Epidemiological studies have shown that individuals with FXI deficiency have lower rates of venous thromboembolism^25^ and ischemic stroke,^26^ with only a modest bleeding tendency. Conversely, elevated FXI plasma levels are associated with increased thrombotic risk: individuals with high FXI levels have more than twice the risk of venous thromboembolism versus the general population.^27^ A Mendelian randomization analysis further supported a causal effect of higher genetically determined FXI levels on ischemic stroke risk (OR 2.54; 95% CI 1.68–3.84; p = 1 × 10^−^^5^), especially cardioembolic stroke (OR 4.23; 95% CI 1.94–9.19).^28^ High FXI activity was also associated with an increased risk of a composite of recurrent stroke, myocardial infarction, or all‐cause death within 3 years after a first ischemic stroke (HR 1.80; 95% CI 1.09–2.98).^29^

FXI also promotes thrombus stability through antifibrinolytic effects. FXIa enhances thrombin generation, which activates thrombin‐activatable fibrinolysis inhibitor (TAFI) and produces fibrin clots more resistant to lysis.^30^, ^31^ In vivo, FXI neutralization results in an almost two‐fold increase in endogenous thrombolysis through reduced TAFI activation.^32^ This may explain why bleeding in FXI deficiency is particularly evident in fibrinolysis‐active tissues, and why FXI inhibition may generate less thrombogenic and more fibrinolysis‐sensitive clots.

Emerging evidence also suggests that FXI exerts biological effects beyond thrombin generation, including contributions to endothelial dysfunction and vascular inflammation.^33^, ^34^, ^35^ Taken together, structural biology, pathophysiology, epidemiology, and preclinical data identify FXI as a key mediator of pathological thrombus amplification and stabilization, while being relatively less essential for routine hemostasis.

Preclinical models have been central in establishing that FXI contributes to ischemic brain injury and that this contribution can be dissociated from physiological hemostasis. In experimental cerebral ischemia, mice deficient in FXI are protected in the transient middle cerebral artery occlusion (tMCAO) model, showing reduced fibrin formation within ischemic vessels, fewer occluded microvessels, and smaller infarct volumes than wild‐type animals.^36^ Genetic deletion, however, does not establish whether the same protection is pharmacologically attainable. This was subsequently demonstrated with 14E11, a monoclonal antibody directed against the apple 2 domain of FXI that selectively prevents FXIIa‐mediated FXI activation while sparing thrombin‐mediated FXI activation, thereby blocking contact‐pathway‐driven thrombus propagation without abolishing the hemostatic function of FXI. In the tMCAO/reperfusion model, 14E11 reduced cerebral infarction and intravascular fibrin deposition, improved cortical reperfusion, and enhanced neurological outcome, with no detectable impairment of hemostasis.^37^

The relevance of FXI in the central nervous system is not confined to intravascular thrombus growth. FXI operates at the interface between coagulation and inflammation, a concept now referred to as thromboinflammation. In murine polymicrobial sepsis, inhibition of FXI activation reduced systemic thrombin generation and circulating interleukin‐6 and tumor necrosis factor‐α, limited platelet consumption, and improved survival without increasing bleeding.^38^ More recently, a non‐coagulant function has been characterized: FXIa promotes ADAM10‐mediated cleavage of the vascular endothelial‐cadherin extracellular domain through VEGFR2 and MAPK signaling, thereby increasing endothelial permeability, while inhibition of FXI activation preserves barrier integrity in vivo.^35^ This mechanism is of direct cerebrovascular interest, since blood‐brain barrier disruption contributes both to the progression of ischemic injury and to hemorrhagic transformation. Consistently, pharmacological FXI inhibition with the same antibody used in the previously mentioned stroke model attenuated experimental autoimmune encephalomyelitis, reducing clinical severity, mononuclear cell infiltration of the brain, axonal damage, and fibrin deposition in the spinal cord.^39^ Overall, the preclinical evidence shows that FXI amplifies and stabilizes pathological thrombus and, in addition, modulates endothelial permeability and neuroinflammation, while remaining largely not essential for hemostasis. These effects distinguish FXI from the targets of currently available anticoagulants and underlies the expectation of a wider therapeutic window in cerebrovascular disease.

## Pharmacology of Factor XI Inhibition

FXI‐directed anticoagulation relies on three principal pharmacological classes: small‐molecule oral direct FXIa inhibitors, antisense oligonucleotides (ASOs), and monoclonal antibodies. Although all three attenuate thrombin amplification within the intrinsic pathway, they act at distinct biological levels and therefore display markedly different pharmacokinetic and pharmacodynamic profiles.^9^ Small molecules inhibit circulating FXIa directly and reversibly; ASOs suppress hepatic FXI biosynthesis; and monoclonal antibodies neutralize FXI/FXIa or prevent FXI activation. These differences are clinically meaningful because they determine the need to establish the appropriate route of administration, effect onset and offset, dosing flexibility, drug‐drug interactions, and practical management of bleeding.^10^

### Small‐Molecule Direct Oral FXIa Inhibitors

Among FXI‐directed agents, the orally active small molecules asundexian and milvexian are at present the most pharmacologically relevant for chronic cerebrovascular prevention, combining oral administration with rapid target engagement and reversible FXIa blockade. The main pharmacological differences are summarized in Table 1.

**Table 1:** Comparative Pharmacological Properties of Asundexian and Milvexian.

Characteristics	Asundexian	Milvexian
	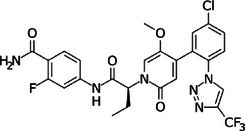	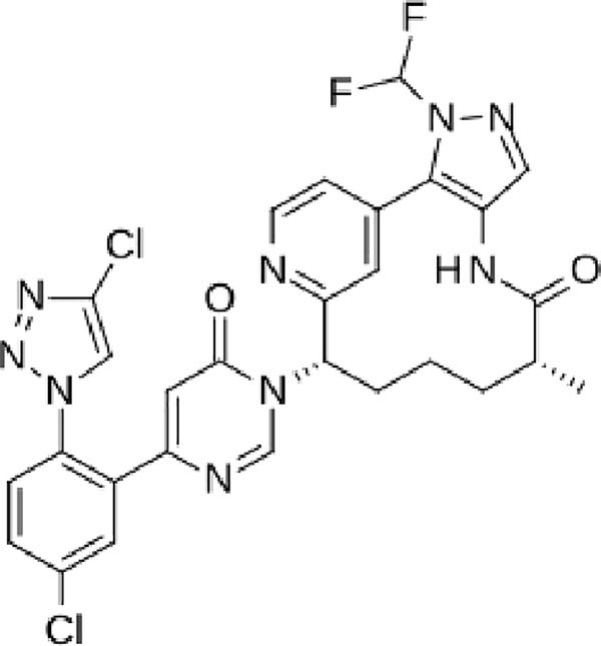
Code/other names	BAY 2433334	BMS‐986177/JNJ‐70033093
Molecular weight	592.9 g/mol	626.4 g/mol
Inhibitory potency (Ki vs human FXIa)	Not available	0.11 nM
Selectivity	>1000‐fold over other serine proteases	>1000‐fold over other serine proteases
**Pharmacokinetics**		
Route of administration	Oral	Oral
Prodrug	No	No
Oral bioavailability (F)	∼94%	58%^a^
t_max_	1–1.5 h (oral solution); 2–4 h (IR tablets)	2.5–4 h (fasted); 7–8 h (after meal, high doses)
Elimination half‐life (t_½_)	∼14–18 h	∼8.3–13.8 h
Apparent volume of distribution at steady state (V_d(ss)_)	∼70.5 L	Not reported
Apparent volume of distribution, terminal phase (V_z_/F)	Not reported	347 L
Apparent oral clearance (Cl/F)	2.56 L/h (CV 20.5%; ≈ 43 mL/min)	18.6 L/h (CV 90.6%; ≈ 310 mL/min)
Plasma protein binding	∼94%	∼92%
Metabolism	CES1 (primary); CYP3A4 (minor)	CYP3A4 (primary)
P‐glycoprotein substrate	Yes	Yes
OATP1B1/1B3 substrate	Not reported	No
Excretion	Fecal ∼80%; urinary ∼20% (6% unmodified)	Fecal (predominant); urinary <20% (7%–18% unmodified)
Food effect	Modest (AUC not clinically affected)	Pronounced at high doses
**Drug‐drug interactions**		
Strong CYP3A4 + P‐gp inhibitors	AUC +103% (itraconazole); +76% (verapamil)	AUC +2.5‐fold (itraconazole)
Moderate CYP3A4 inhibitors	No meaningful effect (fluconazole)	AUC +38% (diltiazem)
CYP3A4 + P‐gp inducers	AUC −44% (carbamazepine)	AUC reduced to ∼15% (rifampin)
**Pharmacodynamics**		
FXIa inhibition	92% at trough/94% at peak (50 mg)	Concentration dependent
Effect on aPTT	Dose‐dependent prolongation	Dose‐dependent prolongation
Effect on PT/INR	No clinically relevant effect	No clinically relevant effect
**Dosing**		
Dosing frequency	Once daily (QD)	Twice daily (BID)
Renal impairment	No dose adjustment required	AUC +41%–54%; no adjustment expected; t_1/2_ prolongation to 18 h
Hepatic impairment	No clinically relevant changes (Child–Pugh A/B)	Modest changes; no adjustment expected
**References**	^42^, ^46^, ^47^, ^48^, ^49^, ^59^	^51^, ^52^, ^54^, ^55^, ^56^, ^57^, ^58^, ^84^, ^112^, ^113^

AUC, area under the concentration–time curve; BID, twice daily; CES1, carboxylesterase‐1; Cl*/*F, apparent oral clearance; CYP, cytochrome P450; FXIa, activated factor XI; IR: immediate release; OATP, organic anion transporting polypeptide; P‐gp, P‐glycoprotein; QD, once daily; t_max_, time to maximum plasma concentration; V_ss_, apparent volume of distribution at steady state; V_z_/F, terminal phase apparent volume of distribution after oral administration.

^a^ After administration of 25 mg milvexian in a spray‐dried dispersion formulation.^84^

Both are low‐molecular‐weight synthetic compounds that bind the catalytic site of FXIa and suppress intrinsic‐pathway amplification without lowering circulating FXI concentrations. In contrast to ASOs and monoclonal antibodies, their pharmacological features such as rapid onset and shorter offset make these molecules feasible for long‐term stroke prevention, particularly in older patients with frequent medication changes, recurrent hospitalizations, or intermittent procedural needs.^40^ The medicinal chemistry programs that led to these compounds were explicitly shaped by the need to balance potency, selectivity, permeability, metabolic stability, and oral exposure, a particularly demanding task for FXIa active‐site inhibitors because of the geometry of the FXIa binding pocket and the need to preserve selectivity over related serine proteases.^41^, ^42^

Preclinical studies have consistently supported the pharmacological rationale of this class. In rabbits and non‐human primates, small‐molecule FXIa inhibition reduced thrombus formation with little or no increase in bleeding time at effective doses with antithrombotic activity. In these studies, earlier compounds (BMS‐262084 and BMS‐654457) and later agents (BMS‐724296) displayed a consistent pattern: dose‐dependent antithrombotic efficacy, prolongation of ex vivo activated partial thromboplastin time (aPTT), no meaningful effect on prothrombin time (PT), and substantially lower bleeding liability than FXa or thrombin inhibitors at comparable antithrombotic effect.^43^, ^44^, ^45^ These data established ex vivo aPTT as a plausible pharmacodynamic surrogate of FXIa target engagement and confirmed that the therapeutic window of FXIa inhibition may be wider than that of common‐pathway anticoagulants.^20^

#### Asundexian Pharmacokinetics

Asundexian demonstrates high oral bioavailability (94%).^46^, ^47^ In phase I studies, exposure increased approximately dose proportionally over the clinically relevant range of 25–100 mg once daily, with low‐to‐moderate variability and minor‐to‐moderate accumulation after repeated dosing.^46^, ^48^ Single doses of 5–150 mg produced dose‐dependent exposure, with a terminal half‐life of 14–18 h, supporting once‐daily administration.^40^, ^46^ Median t_max_ is 1–1.5 h for oral solution and 2–4 h for immediate‐release tablets; after repeated dosing, time‐to‐peak remains consistent with single‐dose values. Food, gastric pH modulation, and tablet formulation had no clinically meaningful effect on total exposure (area under the concentration‐time curve, AUC), whereas high‐caloric meals and antacids modestly reduced or delayed the rate of absorption (C_max_ and t_max_) without altering overall bioavailability. Distribution occurs predominantly within the blood compartment, with an apparent volume of distribution at steady state (V_d(ss)_) of ∼70.5 L.^46^

Asundexian is a low‐clearance compound with negligible first‐pass metabolism.^49^ Human mass‐balance studies demonstrated near‐complete recovery of radioactivity, with elimination mainly in feces (∼80%) and to a lesser extent in urine (∼20%, 6% unmodified). The main clearance pathways are amide hydrolysis and excretion of unchanged parent drug, whereas oxidative biotransformation is a minor pathway.^49^ In plasma, asundexian remains the major drug‐related entity: the main circulating metabolite is the inactive M10.^50^ Asundexian is metabolized predominantly by carboxylesterase 1 (CES1), with a smaller contribution from CYP3A4, and is also a P‐glycoprotein substrate.^46^ Population pharmacokinetic analyses confirmed dose‐proportional pharmacokinetics over 10–100 mg once daily and identified body weight, age, sex, renal function, and concomitant CYP3A4 inhibitors as statistically significant but not clinically relevant covariates, supporting absence of dose adjustment for secondary stroke prevention.^48^

Co‐administration with combined strong CYP3A4/P‐gp inhibitors (itraconazole and verapamil) increased asundexian AUC by 103% and 76%, respectively, whereas moderate CYP3A4 inhibition alone (fluconazole) had no meaningful effect; combined CYP3A4/P‐gp induction by carbamazepine decreased AUC by 44%.^49^ The low‐clearance, high‐bioavailability profile and the predominant role of CES1 rather than CYP3A4 reduce clinically relevant interaction liability relative to more interaction‐sensitive oral anticoagulants. This is a favorable property in stroke patients receiving polytherapy.

#### Milvexian Pharmacokinetics

Milvexian also has a favorable oral profile, with practical differences from asundexian. In phase I studies, milvexian was rapidly absorbed, with t_max_ of 2.5–4 h under fasted conditions.^51^ Elimination half‐life ranged from 8.3–13.8 h after single doses and 11.4–18.1 h after multiple doses,^51^ supporting both once‐ and twice‐daily regimens. In subsequent population studies, the terminal half‐life was 8.9–11.9 h in Japanese participants^49^ and 9–10 h in Chinese participants.^52^, ^53^ Steady state was achieved within ∼6 days of twice‐daily dosing; under fasted conditions, exposure was dose proportional between 25 and 200 mg,^51^ whereas 500 mg with food showed delayed t_max_ (7–8 h) and increased exposure.^54^ These data indicate a predictable pharmacokinetic profile at clinically relevant exposures, albeit more formulation and dose sensitive than that of asundexian.^55^

Milvexian is metabolized mainly via CYP3A4/3A5 and is a substrate of P‐gp; renal excretion is <20% (7%–18% unmodified), and elimination is predominantly hepatobiliary.^51^ This makes milvexian pharmacologically attractive as an oral anticoagulant, but also more vulnerable than asundexian to interaction‐driven variability. In dedicated drug‐drug interaction studies, itraconazole substantially increased milvexian exposure, with an approximately 2.5‐fold increase in AUC and a pronounced increase in trough exposure, whereas diltiazem produced a smaller increase.^56^ Conversely, repeated rifampin administration, reflecting combined CYP3A/P‐gp induction, reduced milvexian exposure, with AUC reduced by ∼85% and C_max_ by ∼78%; in contrast, a single dose of rifampin, reflecting organic anion transporting polypeptide (OATP) inhibition without CYP3A full induction, had little effect, supporting the conclusion that milvexian is a substrate of CYP3A4/5 and P‐gp, but not OATP.^57^ These interaction findings are directly relevant to stroke practice, where enzyme‐inducing antiseizure drugs or strong azole antifungals may alter systemic exposure.

Mild‐to‐moderate hepatic impairment produces only modest exposure changes (AUC geometric mean ratios 1.17 and 1.00, respectively) unlikely to require dose adjustment.^58^ Moderate and severe renal impairment increase AUC by 41% and 54%, respectively, with half‐life prolongation to 18 h, broadly consistent with several current oral anticoagulants.^53^ In Japanese and Chinese participants, the pharmacokinetic/pharmacodynamic profile was similar to non‐Japanese/Caucasian cohorts, with no clinically important ethnic divergence in the concentration‐aPTT relationship.^51^, ^52^, ^54^

These differences explain the practical dosing logic: asundexian, with a half‐life of 14–18 h, linear pharmacokinetics across the clinically relevant range, minimal food effect, and stable once‐daily exposure, is well suited to once‐daily dosing. By contrast, because of the shorter effective half‐life, more pronounced food effect at high doses and greater CYP3A/P‐gp sensitivity, milvexian supports twice‐daily dosing when 24‐h target coverage is desired. This may be clinically useful in stroke prevention, where trough escape, adherence, co‐medication burden, and short‐term treatment interruptions influence long‐term net benefit.

#### Pharmacodynamics

Both asundexian and milvexian produce concentration‐dependent FXIa inhibition with parallel aPTT prolongation, while PT remains essentially unchanged, reinforcing selectivity for the intrinsic pathway. For asundexian, FXIa inhibition is rapid and dose dependent, with effects closely tracking plasma concentrations; in PACIFIC‐AF trial, asundexian 20 mg produced an 81% reduction in baseline FXIa at trough (C_min_) and 90% at peak (C_max_), whereas 50 mg produced 92% at C_min_ and 94% at C_max_.^59^ For milvexian, aPTT prolongation and FXI clotting‐activity reductions closely track plasma exposure, with maximal effects near t_max_.^52^, ^54^ aPTT has served as the most accessible pharmacodynamic biomarker in both programs, and preclinical studies with direct FXIa inhibitors also support ex vivo aPTT as a pharmacodynamically meaningful correlate of antithrombotic activity.^20^ However, no validated exposure, aPTT, or residual FXIa threshold has yet been established to predict efficacy or bleeding in secondary stroke prevention, and this remains a major unresolved issue.

### Antisense Oligonucleotides and Monoclonal Antibodies

ASOs and monoclonal antibodies are pharmacologically distinct from oral FXIa inhibitors because they are parenteral, longer acting, and less flexible for chronic antithrombotic management. The main pharmacological features are summarized in Tables 2 and 3, respectively.

**Table 2:** Comparative Pharmacological Properties of Factor XI‐Targeted Antisense Oligonucleotides (ASOs).

Characteristics	IONIS‐FXIRx	Fesomersen
Code/other names	ISIS‐416858; BAY2306001	IONIS‐FXI‐LRx; FXI‐LICA; BAY2976217
Molecular weight	Not reported; typical of unconjugated 2′‐MOE ASOs (∼7–10 kDa)	Not reported; typical of GalNAc3‐conjugated ASO (∼8–16 kDa)
Drug class	2nd‐generation 2′‐MOE antisense oligonucleotide (unconjugated)	GalNAc3‐conjugated 2′‐MOE antisense oligonucleotide
**Pharmacokinetics**		
Route of administration	Subcutaneous	Subcutaneous
Prodrug	No	No (GalNAc3 moiety cleaved by endolysosomal degradation)^a^
t_max_	∼6 h	∼1.5–3.5 h
Elimination half‐life (t_½_)	∼1–2 weeks (∼168–336 h)	∼10–20 days (∼240–480 h)
Apparent volume of distribution (V_d(SS)_)	Not reported	Not reported
Apparent plasma clearance (Cl/F)	Not reported	Not reported
Plasma protein binding	High (>90%, typical of 2′‐MOE ASOs)	High (>90%, typical of 2′‐MOE ASOs)
Metabolism	Endonuclease/exonuclease‐mediated metabolism; no CYP450 involvement	Endonuclease/exonuclease‐mediated metabolism; no CYP450 involvement
CYP450 substrate	No	No
P‐glycoprotein substrate	No	No
Renal excretion	Minimal (<1% of dose recovered unchanged in urine); low renal elimination	After subcutaneous dosing, ∼25% recovered in urine and ∼72% in feces (radiolabelled GalNAc); predominantly hepatic uptake and catabolism
**Drug–drug interactions**		
Classical PK interactions	Low risk (no CYP metabolism; no P‐gp)	Low risk (no CYP metabolism; no P‐gp)
Pharmacodynamic interactions	Additive bleeding risk with other anticoagulants/antiplatelets	Additive bleeding risk with other anticoagulants/antiplatelets
**Pharmacodynamics**		
Effect on circulating FXI	Dose‐dependent reduction of plasma FXI; maximal effect reached only after prolonged administration (weeks)	In RE‐THINC (patients with kidney failure on hemodialysis), median steady‐state FXI reduction: 53.6% (40 mg), 71.3% (80 mg), 86.0% (120 mg) versus 1.9% with placebo
Effect on aPTT	Dose‐dependent prolongation after prolonged administration	Dose‐dependent prolongation after prolonged administration
Effect on PT/INR	No clinically relevant effect	No clinically relevant effect
Onset / offset	Slow onset (days‐weeks); slow offset (half‐life driven)	Slow onset (days‐weeks); slow offset (half‐life driven)
Reversibility	No specific reversal agent available	No specific reversal agent available
**Dosing**		
Dosing frequency	200–300 mg subcutaneous, weekly (FXI‐ASO TKA trial)	40–120 mg subcutaneous, monthly (RE‐THINC trial)
Renal impairment	Evaluated in ESRD on hemodialysis; no dose adjustment anticipated; low renal elimination	Evaluated in ESRD on hemodialysis (RE‐THINC); no dose adjustment anticipated
Hepatic impairment	No dedicated studies	No dedicated studies; hepatic uptake relevant for GalNAc‐ASO, clinical impact unclear
**References**	^20^, ^60^, ^61^, ^114^, ^115^	^61^, ^62^, ^114^, ^115^

aPTT, activated partial thromboplastin time; ASGPR, asialoglycoprotein receptor; AUC, area under the concentration‐time curve; Cl/F, apparent plasma clearance; CYP, cytochrome P450; ESRD, end‐stage renal disease; FXI, factor XI; FXIa, activated factor XI; GalNAc3, triantennary N‐acetylgalactosamine; 2′‐MOE, 2′‐O‐methoxyethyl; P‐gp, P‐glycoprotein; PT, prothrombin time; RES, reticuloendothelial system; SC, subcutaneous; TKA, total knee arthroplasty; t_max_, time to maximum plasma concentration; V_d_, apparent volume of distribution; VTE, venous thromboembolism.

^a^ Although the GalNAc3 moiety is cleaved intracellularly by endo/lysosomal degradation following hepatocyte uptake, it functions as a hepatocyte‐targeting ligand rather than a bioreversible prodrug group, and fesomersen is therefore not classified here as a prodrug in the conventional sense. Some authors, however, frame GalNAc‐conjugated oligonucleotides as requiring intracellular processing of the targeting ligand to reach their fully active form.^116^

**Table 3:** Comparative Pharmacological Properties of Factor XI/XIa‐Directed Monoclonal Antibodies.

Characteristics	Abelacimab	Osocimab	BAY1831865	SHR‐2004	REGN7508Cat	REGN9933A2
Code/other names	MAA868	BAY1213790	—	—	—	—
Molecular weight	∼150 kDa	∼150 kDa	∼150 kDa	∼150 kDa	∼150 kDa	∼150 kDa
Format	Fully human IgG	Fully human IgG	Humanized IgG	Humanized IgG	Fully human IgG	Fully human IgG
Target/epitope	FXI/FXIa catalytic domain	FXIa active‐site adjacent region	FXI	FXI and FXIa	FXI catalytic domain	FXI apple 2 domain
**Pharmacokinetics**						
Route of administration	IV and SC	IV and SC	IV and SC	IV and SC	IV (reported in ROXI‐VTE‐II)	IV (reported in ROXI‐VTE‐I)
Dosing in trials	150 mg IV loading + monthly SC; 90 or 150 mg monthly SC (AZALEA‐TIMI 71)	0.3, 0.6, 1.2, 1.8 mg/kg IV single dose (FOXTROT)	Single‐dose escalation (phase I)	Phase I dose escalation	250 mg IV post‐surgery single dose (ROXI‐VTE‐II)	300 mg IV post‐surgery single dose (ROXI‐VTE‐I)
Frequency	Monthly SC (after IV load)	Monthly or peri‐procedural IV/SC	Single dose (phase I)	Single dose (phase I)	Single‐dose IV (ROXI‐VTE‐II)	Single dose (ROXI‐VTE‐I)
Bioavailability (F)	85% (SC)	75%–85% (SC)	47.2% (95% CI 30.2–73.7) (SC)	61% (SC)	Not reported	Not reported
t_max_	∼1 h (IV); ∼7–21 days (∼168–504 h) (SC)	∼1–3 h (IV); ∼4–6 days (∼96–144 h) (SC)	1.0–2.0 h (IV); 96 h (SC)	∼96–168 h (SC)	Not reported	Not reported
Elimination half‐life (t_½_)	25–30 days (∼600–720 h)	30–44 days (∼720–1056 h)	1.2–8.7 days (28–208 h) (dose dependent); duration of effect up to ∼55 days (∼1320 h)	11.6–13.0 days (∼278–312 h)	Not reported	Not reported
Volume of distribution	V_d(SS)_ 8.48 L (IV); 10.5 L (SC)	V_d(SS)_ 5.50–10.3 L (IV); V_d(area)_/F ∼13.3–16.5 L (SC)	V_d(SS)_ 3.36–4.67 L IV; V_d(area)_/F ∼ 9.10 L SC	V_d(area)_ ∼ 3.84–4.61 L (IV); V_d(area)_/F 5.58–6.85 L (SC)	Not reported	Not reported
Metabolism	Proteolysis by the RES	Proteolysis by the RES	Proteolysis by the RES	Proteolysis by the RES	Proteolysis by the RES	Proteolysis by the RES
Clearance	Cl_B_ IV: 0.0102 L/h (0.17 mL/min); SC: *Cl*/F 0.0124 L/h (0.21 mL/min)	Cl_B_ IV: 6.41–13.5 mL/h (0.11–0.23 mL/min); SC: Cl/F 10.8–15.8 mL/h	Cl_B_ dose dependent: IV 0.0815 L/h (7 mg) → 0.0137 L/h (150 mg); SC Cl/F 0.0291 L/h	Cl_B_ IV: 0.206–0.267 L/day (8.6–11.1 mL/h); SC: Cl/F 0.330–0.407 L/day	Not reported	Not reported
Renal/hepatic clearance	Not renally or hepatically eliminated	Not renally or hepatically eliminated	Not renally or hepatically eliminated	Not renally or hepatically eliminated	Not renally or hepatically eliminated	Not renally or hepatically eliminated
CYP450/P‐gp substrate	No	No	No	No	No	No
Immunogenicity (ADA)	No ADA detected after IV or SC doses	ADA in 6/56 volunteers (Phase I)	Not reported	Not reported	Not reported	Not reported
**Pharmacodynamics**						
Effect on free FXI	>99% reduction within 1 h of IV infusion; 99% (150 mg) and 97% (90 mg) at 3 months with monthly SC (AZALEA)	FXI activity reduction sustained over weeks after single dose	FXI activity reduction with duration up to ∼55 days	Concentration‐dependent FXI/FXIa inhibition	Blocks FXIa activity and FXI activation	Blocks FXIa activity and FXI activation
Effect on aPTT	Dose‐dependent prolongation	Dose‐dependent prolongation	Dose‐dependent prolongation	Dose‐dependent prolongation	Dose‐dependent prolongation	Dose‐dependent prolongation
Effect on PT/INR	No clinically relevant effect	No clinically relevant effect	No clinically relevant effect	No clinically relevant effect	No clinically relevant effect	No clinically relevant effect
**References**	^66^, ^67^, ^69^, ^114^	^61^, ^70^, ^71^, ^115^	^68^, ^114^	^72^	^63^	^63^

ADA, anti‐drug antibodies; aPTT, activated partial thromboplastin time; Cl_B_, total body (plasma) clearance after intravenous administration; Cl/F, apparent clearance after subcutaneous administration; CYP, cytochrome P450; ESRD, end‐stage renal disease; FXI, factor XI; FXIa, activated factor XI; IgG, immunoglobulin G; IV, intravenous; P‐gp, P‐glycoprotein; PT, prothrombin time; RES, reticuloendothelial system; SC, subcutaneous; TKA, total knee arthroplasty; t_max_, time to maximum plasma concentration; V_d(area)_/F, apparent volume of distribution during the terminal phase after extravascular administration; V_d(SS)_, apparent volume of distribution at steady state; VTE, venous thromboembolism.

ASOs such as IONIS‐FXIRx and fesomersen target hepatic FXI mRNA, reducing FXI synthesis rather than directly inhibiting FXIa. They are administered subcutaneously, with elimination half‐lives of approximately 1–2 weeks for IONIS‐FXIRx and 10–20 days for fesomersen, with maximal aPTT prolongation achieved only after prolonged administration.^20^, ^60^ Fesomersen, the GalNAc‐conjugated successor of IONIS‐FXIRx, targets hepatocyte uptake via the asialoglycoprotein receptor,^61^ permits lower doses and allows monthly rather than weekly dosing. IONIS‐FXIRx exhibits high plasma protein binding (>90%) and minimal renal excretion, with <1% of the administered dose recovered unchanged in urine, consistent with low renal elimination. Fesomersen similarly exhibits high plasma protein binding (>90%); after subcutaneous administration, approximately 25% of the dose was recovered in urine and 72% in feces, indicating predominantly hepatic uptake and catabolism. In the phase IIb trial RE‐THINC in patients with kidney failure on hemodialysis, fesomersen produced dose‐dependent steady‐state reductions in median FXI level of 53.6% (40 mg), 71.3% (80 mg), and 86.0% (120 mg) versus 1.9% with placebo.^62^ Because ASOs are metabolized via nuclease‐mediated degradation rather than CYP450 pathways, they carry a low risk of CYP‐based interactions.

Monoclonal antibodies, such as abelacimab and osocimab, and the investigational agents BAY1831865, SHR‐2004, REGN9933A2, and REGN7508Cat are administered intravenously or subcutaneously and produce rapid target engagement with prolonged effect.^60^, ^63^ Four are fully human IgG (abelacimab, osocimab, REGN7508, and REGN9933) and two are humanized IgG (BAY1831865 and SHR‐2004), targeting distinct epitopes on FXI/FXIa as detailed in Table 3. Terminal half‐lives, however, are heterogeneous across compounds: ∼25–30 days for abelacimab, ∼30–44 days for osocimab, ∼11.6–13.0 days for SHR‐2004, and 1.2–8.7 days (28–208 h, dose dependent) for BAY1831865, the latter nonetheless maintaining a duration of FXI inhibition up to ∼55 days.^40^ These agents are metabolized predominantly through proteolysis within the reticuloendothelial system rather than hepatic CYPs or renal clearance, minimizing classical pharmacokinetic interactions and potentially offering advantages in severe renal dysfunction.^64^ The counterbalance is slow reversibility: infrequent dosing is possible but these agents are less adaptable to urgent surgery, trauma, or major bleeding.^65^

Data on immunogenicity of FXI‐targeting monoclonal antibodies remain limited. For abelacimab, no anti‐drug antibodies (ADA) were detected after single intravenous dosing in healthy volunteers or after repeated monthly subcutaneous administration in patients with atrial fibrillation.^66^, ^67^ For osocimab, ADA were detected in 6 of the 56 healthy volunteers (10.7%) in phase I studies.^61^ For BAY1831865, no hypersensitivity or infusion‐related reactions were reported in phase I^68^; specific ADA data have not been published. For SHR‐2004, REGN7508Cat, and REGN9933A2, immunogenicity data have not been reported in available publications. Long‐term immunogenicity assessment remains necessary for agents intended for chronic subcutaneous administration.

Abelacimab is a fully human monoclonal antibody that binds the catalytic domain of FXI, locking it in the inactive zymogen conformation and preventing activation by both FXIIa and thrombin.^66^ Following intravenous administration, abelacimab has a terminal half‐life of 25–30 days, with >99% reductions in free FXI within 1 h of infusion.^67^ In AZALEA‐TIMI 71 trial in atrial fibrillation patients, monthly subcutaneous abelacimab 150 or 90 mg produced, respectively, sustained median free‐FXI reductions of 99% and 97% at 3 months.^69^ The trial was stopped early for a greater‐than‐anticipated bleeding reduction versus rivaroxaban (HR 0.38 for 150 mg; 0.31 for 90 mg; *P* <.001 for both).

Osocimab is a fully human monoclonal antibody binding the adjacent region to the active site of FXIa and preventing FIX activation. After intravenous infusion in healthy volunteers, osocimab has a half‐life of 30–44 days, enabling single‐dose administration for surgical thromboprophylaxis.^70^ Phase 1 studies in healthy East Asian volunteers showed a pharmacokinetic/pharmacodynamic profile similar to Caucasian populations, with no clinically relevant ethnic differences.^71^ In the FOXTROT trial, preoperative osocimab 1.8 mg/kg was superior to enoxaparin for venous thromboembolism prevention after knee arthroplasty (11.3% vs 26.3%; *P* = .007 for superiority).^70^

BAY1831865 is a humanized FXI‐directed monoclonal antibody with terminal half‐life that increases with dose (range 28–208 h), with duration of effect up to 55 days.^68^ SHR‐2004 is a humanized monoclonal antibody selectively binding FXI and FXIa, with mean half‐life 11.6–13.0 days.^72^ REGN7508Cat binds the FXI catalytic domain and blocks both its activity and activation by FXIIa and thrombin, whereas REGN9933A2 binds the FXI apple 2 domain and blocks FXI activation by FXIIa only. In the ROXI‐VTE‐II trial, REGN7508Cat was superior to enoxaparin for venous thromboembolism prevention (7% vs 17%), whereas REGN9933A2 was not superior to enoxaparin in ROXI‐VTE‐I trial.^63^

### Clinical Implications of the Pharmacokinetic Differences Among FXI Inhibitors

The most clinically relevant distinction lies between chronic oral FXIa inhibition and long‐acting parenteral FXI‐lowering strategies. Oral agents such as asundexian and milvexian offer rapid onset, shorter half‐lives, and easier temporary interruption, features especially relevant in secondary stroke prevention where patients are often elderly, frail, and exposed to frequent medication changes, intercurrent illness, or invasive procedures.^40^, ^73^ Their principal limitation is potential CYP3A/P‐gp‐mediated variability, especially with antiarrhythmics, antifungals, enzyme‐inducing antiseizure drugs or other cardiovascular therapies. By contrast, ASOs and monoclonal antibodies provide stable, durable FXI suppression, infrequent dosing, and lower burden of classical metabolic interactions, at the cost of slower reversibility and more difficult periprocedural management.^65^, ^74^

The asymmetry in reversibility between short‐ and long‐acting agents deserves particular emphasis, because it is likely to become a principal determinant of clinical implementation rather than a secondary pharmacological detail. Oral small molecules lose their effect within hours of discontinuation, whereas ASOs and monoclonal antibodies suppress FXI for weeks to months, and this cannot be meaningfully shortened: in a patient presenting with intracranial hemorrhage, major trauma, or the need for emergency neurosurgery, no strategy is currently available to restore FXI activity rapidly. This is especially pertinent in stroke patients, in which spontaneous ICH, hemorrhagic transformation, falls, and urgent procedures are more frequent than in the general population.

These differences also affect bleeding management. Short‐acting oral FXIa inhibitors retain a practical advantage over long‐acting parenteral FXI‐lowering therapies. For ASOs, FXI concentrate rapidly reverses the anticoagulant effect in preclinical studies; for monoclonal antibodies and small molecules, antifibrinolytic agents (tranexamic acid) and low‐dose recombinant FVIIa may be considered in emergencies, based on experience in congenital FXI deficiency, though availability and potential thrombotic risks remain limitations.^65^ The modest bleeding observed with FXIa inhibitors in preclinical and early clinical studies should not be interpreted as equivalent to zero hemostatic liability, but rather as suggesting a more favorable benefit‐risk balance than common anticoagulants in appropriately selected patients.^63^, ^66^, ^70^

Overall, while all three classes support the broader concept of hemostasis‐sparing anticoagulation, small‐molecule oral direct FXIa inhibitors currently appear to be the most adaptable platform for long‐term secondary prevention after ischemic stroke.

## Clinical Evidence on Factor XI Inhibition for Ischemic Stroke Secondary Prevention

The clinical development of FXI‐directed therapy has moved into indication‐specific evaluation, but the evidence for ischemic stroke prevention remains limited to a small number of drugs and trials. The field is currently driven by the two oral small‐molecule FXIa inhibitors, asundexian and milvexian, which have completed phase II studies in recent ischemic stroke or high‐risk transient ischemic attack (TIA), and are now being evaluated in dedicated phase III programs.

In stroke prevention, FXI inhibitors may have value in at least three contexts. First, they may provide an alternative to standard anticoagulation in atrial fibrillation, where cardioembolic stroke prevention is highly effective with DOACs but still limited by bleeding concerns, particularly in frail and high‐risk patients. Second, FXI inhibition may be relevant in secondary prevention after non‐cardioembolic ischemic stroke, especially in patients with persistent thrombotic risk despite maximal antiplatelet therapy. Third, FXI inhibition may be particularly useful in thrombotic settings such as extracorporeal circuits, dialysis systems, catheters, or implanted devices, where cerebral embolic complications may occur and conventional anticoagulation often increases bleeding liability.

### Results of Randomized Clinical Trials Focused on Stroke Prevention

Design features and key findings of the principal RCTs evaluating asundexian and milvexian in secondary ischemic stroke prevention are summarized in Table 4.

**Table 4:** Phase II and III FXIa Inhibitor Trials for the Prevention of Ischemic Stroke

	Phase II Trials		Phase III Trials	
**Trial**	PACIFIC‐Stroke (NCT04304508)	AXIOMATIC‐SSP (NCT03766581)	OCEANIC‐STROKE (NCT05686070)	LIBREXIA‐STROKE (NCT05702034)
**Drug**	Asundexian	Milvexian	Asundexian	Milvexian
**Dose**	10, 20, or 50 mg PO once daily	25 mg QD or 25, 50, 100, 200 mg PO BID	50 mg PO once daily	25 mg PO BID
**Comparator**	vs placebo; on top of standard SAPT or DAPT	vs placebo; all patients received clopidogrel 75 mg for 21 days + aspirin 100 mg for 90 days	vs placebo; on top of planned SAPT or DAPT	vs placebo; on top of standard SAPT or DAPT
**Time from index event**	Within 48 h	Within 48 h	Within 72 h	Within 48 h
**Main eligibility criteria**	Age ≥45 years • Recent non‐cardioembolic ischemic stroke • NIHSS ≤7 in part A; ≤15 in part B • MRI feasible at baseline/within 72 h	Age ≥40 y • Acute non‐lacunar ischemic stroke (NIHSS ≤5, later ≤7) or high‐risk TIA (ABCD^2^ ≥6) Evidence of visible intracranial or extracranial atherosclerotic plaque, ulceration, or thrombus of any degree without occlusion in a feeding artery	Age ≥18 y Non‐cardioembolic ischemic stroke (NIHSS ≤15) or high‐risk TIA (ABCD^2^ 6–7) • At least one atherosclerotic feature: cerebrovascular atherosclerotic plaque on vascular imaging, medical history of systemic atherosclerosis (CAD/AMI, PAD, carotid stenosis ≥50%, prior carotid revascularization, or aortic plaque), or acute non‐lacunar infarct on imaging (cortical and/or >20 mm on DWI MRI or >15 mm on CT); • Excluded stroke sources requiring anticoagulation and active non‐trivial bleeding	Adults ≥40 y • Acute non‐cardioembolic ischemic stroke (NIHSS ≤7) or high‐risk TIA (ABCD^2^ ≥6) • Early adjunctive treatment strategy on background antiplatelet therapy
**N randomized**	1808	2366	12,327	Ongoing; estimated enrollment 15,000
**Primary efficacy outcome**	Dose‐response for composite of recurrent symptomatic ischemic stroke + MRI covert brain infarction by 26 weeks	Dose‐response for composite of symptomatic ischemic stroke + MRI covert brain infarction at 90 days	Time to first ischemic stroke	Time to first ischemic stroke
**Main results**	Neutral primary efficacy analysis Primary outcome: 19% placebo versus 19% (10 mg), 22% (20 mg), and 20% (50 mg) For asundexian 50 mg versus placebo IR 1.06 (90% CI 0.85–1.32); no significant dose–response (*P* = .80)	Neutral primary efficacy analysis Primary outcome: 16.8% with placebo versus 16.7% (25 mg QD), 16.6% (25 mg BID), 15.6% (50 mg BID), 15.4% (100 mg BID), and 15.3% (200 mg BID) No significant dose‐response was observed Model‐based relative risks versus placebo ranged from 0.99 (25 mg QD) to 0.91 (200 mg BID)	Positive trial; • Asundexian significantly reduced ischemic stroke versus placebo (6.2% versus 8.4%; HR 0.74, 95% CI 0.65–0.84; *P* <.001), with a 26% relative risk reduction All strokes were also reduced (6.6% versus 8.8%; HR 0.74, 95% CI 0.65–0.84; *P* <.001)	Ongoing study; no efficacy results available yet
**Safety**	Major or CRNM bleeding not significantly increased: pooled HR 1.57 (90% CI 0.91–2.71) • New cerebral microbleeds 10.2% versus 10.5% (OR 0.96; 95% CI 0.66–1.41) • No excess hemorrhagic transformation	Major bleeding at 90 days infrequent: 1% placebo, 1% (25 mg QD), 1% (25 mg BID), 2% (50 mg BID), 2% (100 mg BID), 1% (200 mg BID); no apparent dose‐response Any bleeding: 8% placebo versus 9%–13% with milvexian; mostly non‐major GI bleeding at ≥50 mg BID	Major bleeding occurred in 1.9% with asundexian and 1.7% with placebo (HR 1.10, 95% CI 0.85–1.44), indicating no significant increase in major bleeding There was also no excess in symptomatic ICH, hemorrhagic stroke, fatal bleeding, or CRNM bleeding	Ongoing study; no safety results available yet
**Reference**	^73^	^83^	^79^	^84^

AMI, acute myocardial infarction; BID, twice daily; CAD, coronary artery disease; CI, confidence interval; CRNM, clinically relevant non‐major; CT, computed tomography; DAPT, dual antiplatelet therapy; DWI, diffusion‐weighted imaging; GI, gastrointestinal; HR, hazard ratio; ICH, intracranial hemorrhage; IR, incidence ratio; MRI, magnetic resonance imaging; NIHSS, National Institutes of Health Stroke Scale; OR, odds ratio; PAD, peripheral artery disease; PO, per os (oral administration); QD, once daily; SAPT, single antiplatelet therapy; TIA, transient ischemic attack.

PACIFIC‐Stroke (NCT04304508) was an international, randomized, double‐blind, placebo‐controlled phase IIb trial that enrolled 1808 patients aged ≥45 years with recent non‐cardioembolic ischemic stroke within 48 h of symptom onset (National Institute of Health Stroke Scale [NIHSS] ≤7 in Part A and ≤15 in Part B), testing asundexian 10, 20, or 50 mg per os once daily or placebo on top of standard antiplatelet therapy.^73^ The study population reflected the setting in which FXIa inhibition is conceptually most attractive: early secondary prevention after mild‐to‐moderate non‐cardioembolic stroke on single or dual antiplatelet therapy with a narrow therapeutic window.

The primary efficacy analysis was neutral: no significant dose‐response was observed for the composite of recurrent symptomatic ischemic stroke and magnetic resonance imaging (MRI)‐detected covert brain infarction. Event rates were similar across placebo and active arms (19% placebo vs 20% asundexian 50 mg); the 50 mg dose did not establish a significant reduction (IR 1.06; 90% CI 0.85–1.32; t statistic −0.68; *P* for dose‐response = 0.80). Results were driven by covert infarctions (∼75% of events), which may have masked a possible effect on clinically relevant recurrent strokes. A subsequent MRI analysis showed that asundexian 50 mg was associated with a trend toward lower risk of recurrent ischemic stroke or incident covert infarction (HR 0.71; 95% CI 0.45–1.11), that was not evident in patients with single small subcortical infarcts (HR 1.14; 95% CI 0.62–2.10), suggesting that the benefit may be confined to patients with large, cortical or multiple baseline infarcts.^75^ These data may possibly reflect a greater contribution of large‐artery or embolic mechanisms, more biologically relevant to FXIa inhibition than small‐vessel strokes. These exploratory findings were insufficient to redefine the main results, but suggest that patient and endpoint selection were major determinants of apparent treatment neutrality.

The safety findings were more consistently favorable than the efficacy ones: major or clinically relevant non‐major bleeding did not significantly increase with asundexian versus placebo (pooled HR 1.57; 90% CI 0.91–2.71).^73^ A pooled analysis of three phase II asundexian trials (in atrial fibrillation, recent acute coronary syndrome [ACS], or stroke) confirmed bleeding rates similar to placebo on background antiplatelet therapy.^76^ New cerebral microbleeds (CMBs) during follow‐up occurred at virtually identical rates with asundexian and placebo (10.2% vs 10.5%; OR 0.96; 95% CI 0.66–1.41), indicating that their appearance reflects the natural history of the underlying small‐vessel disease rather than a drug effect: in fact, microbleeds were already present on 35% of baseline MRI examinations.^77^ Hemorrhagic transformation was frequently detected on MRI (28% at baseline, 24% at follow‐up) and associated with larger infarcts, cortical involvement, microbleeds, and cortical superficial siderosis, without evidence that FXIa inhibition aggravated this profile.^78^

Phase II results have been determinant to design the phase III OCEANIC‐STROKE trial (NCT05686070), an event‐driven, double‐blind, placebo‐controlled trial of asundexian 50 mg once daily versus placebo on top of single or dual antiplatelet therapy in patients with non‐cardioembolic mild‐to‐moderate ischemic stroke (NIHSS ≤15) or high‐risk TIA defined by ABCD2 score of 6–7 within 72 h of onset.^79^ The primary efficacy outcome was time to ischemic stroke, rather than a composite outcome, and the trial specifically targeted patients with atherosclerotic features. Between January 2023 and February 2025, 12,327 participants were randomized; 95% had ischemic stroke as index event, 43% had large‐artery atherosclerosis, and 63% received dual antiplatelet therapy.

The trial met both primary endpoints^79^: over a median follow‐up of 567 days, ischemic stroke occurred in 6.2% of the asundexian group and 8.4% of the placebo group (cause‐specific HR 0.74; 95% CI 0.65–0.84; *P* <.001); these are overall cumulative incidences over the whole trial. The corresponding 1‐year cumulative incidences estimated by the Aalen‐Johansen method were 5.1% and 7.0%, yielding an absolute risk reduction of 1.9% at 1 year, corresponding to a number needed to treat (NNT) of approximately 53 patients treated for 1 year to prevent one additional ischemic stroke. The treatment effect was consistent across pre‐specified subgroups including age, sex, index event type, NIHSS, etiological subtype, acute reperfusion treatment, and planned single versus dual antiplatelet therapy. Asundexian also significantly reduced all stroke (6.6% vs 8.8%; cause‐specific HR 0.74; 95% CI 0.65–0.84; *P* <.001), the composite of cardiovascular death/myocardial infarction/stroke (HR 0.83; 95% CI 0.74–0.92; *P* <.001), the composite of all‐cause mortality/myocardial infarction/stroke (HR 0.85; 95% CI 0.77–0.95; *P* = .003), and disabling or fatal stroke (HR 0.69; 95% CI 0.55–0.87; ∼31% relative risk reduction). Regarding safety, there was no significant increase in major bleeding (1.9% vs 1.7%; cause‐specific HR 1.10; 95% CI 0.85–1.44) and no excess across any secondary safety endpoints including clinically relevant non‐major bleeding, symptomatic ICH, hemorrhagic stroke, or fatal bleeding.^79^

In OCEANIC‐AF (NCT05643573), asundexian 50 mg once daily in high‐risk patients with atrial fibrillation (mean age 73.9 years; CHA_2_DS_2_‐VASc 4.3; 18.2% prior stroke/TIA) was associated with higher stroke or systemic embolism than apixaban (1.3% vs 0.4%; HR 3.79; 95% CI 2.46–5.83), despite lower major bleeding (0.2% vs 0.7%; HR 0.32; 95% CI 0.18–0.55), leading to early termination.^80^

The divergent outcomes between OCEANIC‐STROKE (positive) and OCEANIC‐AF (negative) highlight that FXI inhibition is highly context dependent: in secondary stroke prevention, where the comparator is placebo and the background is antiplatelet therapy, adding lower FXIa inhibition appears to provide net benefit, whereas in atrial fibrillation, where the comparator is a highly effective DOAC, FXI inhibition alone is insufficient to prevent cardioembolic stroke. Proposed explanations for the latter failure include: (i) FXIa inhibition may not be as effective as apixaban for stroke prevention in this clinical setting; (ii) the 50 mg once‐daily dose may inadequately suppress FXI activity; and (iii) alternative thrombin‐generation pathways that bypass FXI, such as those mediated by tissue factor and/or kallikrein, especially in patients transitioning from ongoing anticoagulation.^81^, ^82^

The second major oral FXIa inhibitor in stroke trials is milvexian. AXIOMATIC‐SSP (NCT03766581) was a randomized, placebo‐controlled, dose‐finding phase II study in 2366 patients aged ≥40 years with acute non‐lacunar ischemic stroke (NIHSS ≤5, later ≤7) or high‐risk TIA within 48 h.^83^ Participants received placebo or milvexian 25 mg once daily or 25, 50, 100, or 200 mg twice daily. All received clopidogrel 75 mg for 21 days and aspirin 100 mg for 90 days, making this a formal test of FXIa inhibition added to dual antiplatelet therapy in early secondary prevention.

Like PACIFIC‐Stroke, AXIOMATIC‐SSP did not show a significant reduction in its primary composite of symptomatic ischemic stroke *plus* MRI covert brain infarction, nor a clear dose‐response: estimated incidence was 16.8% (90.2% CI 14.5–19.1) with placebo versus 16.7%–15.3% across milvexian arms, with model‐based relative risks ranging from 0.99 (25 mg once daily) to 0.91 (200 mg twice daily).^83^ In a secondary analysis, however, milvexian was associated with fewer symptomatic ischemic strokes than placebo at all doses except 200 mg twice daily (placebo 5% [38/691]; 25 mg BID 4% [12/318]; 50 mg BID 4% [13/328]; 100 mg BID 4% [11/310]; 200 mg BID 8% [27/351]), whereas the composite endpoint remained neutral. Notably, the risk ratio of symptomatic ischemic stroke with milvexian 100 mg twice daily was 0.65 (95% CI 0.33–1.25), close to the pre‐specified 32% relative risk ratio considered clinically meaningful, and essentially superimposable on that observed at 25 mg twice daily (RR ∼0.68). This suggests a *plateau* of antithrombotic efficacy already reached at 25 mg BID, beyond which dose escalation did not further reduce stroke but increased non‐major bleeding. These results also suggest that covert infarction, which contributed 66% (227/343) of primary outcome events, may have masked a possible symptomatic signal and that greater FXIa inhibition is not necessarily better in the setting of dual antiplatelet therapy.

The safety profile of milvexian in AXIOMATIC‐SSP was again more reassuring than the efficacy profile. The primary safety endpoint of major and/or fatal bleeding was infrequent and showed no dose–response: 1% with placebo (4/682) and 1%–2% across all milvexian arms. ICH, including symptomatic hemorrhagic transformation, occurred in two placebo‐treated participants (<1%), three receiving 50 mg BID (1%) and one receiving 200 mg BID, with no fatal events. A modest increase in non‐major, mostly gastrointestinal bleeding was observed at ≥50 mg BID; any type bleeding occurred in 8% (54/682) of placebo patients versus 9%–13% across milvexian arms, with most events by day 21.^83^

The ongoing phase III LIBREXIA‐STROKE trial (NCT05702034) reflects a much more pharmacology‐guided strategy: the selected stroke dose is milvexian 25 mg twice daily, which provided the most favorable balance between recurrent symptomatic stroke signal and bleeding risk in AXIOMATIC‐SSP. The trial enrolls adults ≥40 years with acute ischemic stroke (NIHSS ≤7) or high‐risk TIA randomized within 48 h. Dose selection was also informed by prior dual‐pathway inhibition experience showing that when anticoagulation is added to antiplatelet therapy, lower anticoagulant intensity may yield a better benefit–risk profile than full‐intensity dosing.^84^ The same 25 mg BID dose was selected for LIBREXIA‐ACS (NCT05754957), a phase III trial in recent ACS patients stopped in November 2025 at *ad interim* analysis for futility.^85^ Otherwise, the 25 mg BID dose is four‐fold lower than the 100 mg BID regimen chosen for LIBREXIA‐AF (NCT05757869),^86^ in which milvexian is compared head‐to‐head with apixaban in patients with atrial fibrillation. The lower dose in stroke and ACS reflects the fact that both secondary stroke prevention and post‐ACS setting differ fundamentally from the atrial fibrillation one, because the comparator is placebo and the background therapy is single or dual antiplatelet treatment, so that lower anticoagulant intensity is expected to preserve antithrombotic efficacy while minimizing additive bleeding risk. This is further supported by prior dual‐pathway inhibition experience: in ATLAS ACS 2‐TIMI 51 (NCT00809965), low‐dose rivaroxaban (2.5 mg BID) on top of antiplatelet therapy improved outcomes in ACS, whereas full‐dose apixaban (5 mg BID) added to antiplatelet therapy in APPRAISE‐2 (NCT00831441) was stopped early for excess bleeding, including ICH.^84^ LIBREXIA‐STROKE is therefore testing a specific hypothesis about low‐intensity oral FXIa inhibition as adjunct to antiplatelet therapy. The trial is in its final stages and results are expected by the end of 2026.

Data from these trials nonetheless require extensive clinical validation, because the populations studied were markedly heterogeneous. All stroke programs restricted enrollment by stroke severity but with different NIHSS thresholds, and differed also in randomization window, qualifying TIA definition, requirement for atherosclerotic features and background antiplatelet regimen. Two consequences follow: first, the enrolled cohorts represent only a subgroup of the real‐world stroke population (mild‐to‐moderate deficits, early randomization, and no indication for anticoagulation), whereas patients with severe stroke, large infarcts, or high risk of hemorrhagic transformation, who carry the highest absolute risk of both recurrence and bleeding, remain unstudied. Second, this heterogeneity makes cross‐trial comparison hazardous.

### Synthesizing the Evidence: Meta‐Analytic Results on FXI/XIa Inhibitors for Ischemic Stroke Prevention

Several systematic reviews and meta‐analyses have synthesized the rapidly expanding FXI/XIa evidence, although heterogeneity across clinical settings, drug classes, comparators, and background antithrombotic regimens limit direct cross‐study comparison.

The first stroke‐dedicated meta‐analysis by Palaiodimou et al^87^ pooled four phase II trials (including PACIFIC‐Stroke and AXIOMATIC‐SSP; 4,732 FXIa‐treated patients vs 1798 controls) and reported a non‐significant trend toward reduced ischemic stroke recurrence (RR 0.89; 95% CI 0.67–1.17), becoming borderline when TIA was added (RR 0.78; 95% CI 0.61–1.01). Major or clinically relevant non‐major bleeding was not significantly increased (RR 1.19; 95% CI 0.65–2.16) and ICH was numerically similar (RR 0.91; 95% CI 0.26–3.19), supporting a favorable safety indication in secondary stroke prevention.

Galli et al^88^ meta‐analyzed eight phase II RCTs (n = 9216) across orthopedic, atrial fibrillation, stroke, and ACS settings. Against low‐molecular‐weight heparin, FXI inhibitors reduced any bleeding (RR 0.49; 95% CI 0.31–0.77) and thrombotic endpoints (RR 0.62; 95% CI 0.49–0.79), whereas in placebo‐controlled stroke (PACIFIC‐Stroke, AXIOMATIC‐SSP) and post‐myocardial infarction (PACIFIC‐AMI) settings the efficacy endpoint was unchanged (RR 1.02; 95% CI 0.9–1.13), with only a dose‐dependent increase in minor bleeding. Franco‐Moreno et al^89^ reached concordant conclusions, describing the phase II stroke trials as showing modest efficacy with preserved safety.

The meta‐analysis by Zahid et al,^90^ which pooled 14 RCTs including 30,952 patients, confirmed that FXI/XIa inhibitors significantly reduce major bleeding compared with controls (RR 0.47; 95% CI 0.33–0.66), with a trend toward reduced ICH that did not reach statistical significance (RR 0.49; 95% CI 0.23–1.05). Across all indications, the effect on ischemic stroke was neutral (RR 0.99; 95% CI 0.49–1.48). However, in the atrial fibrillation subgroup, FXI/XIa inhibitors were associated with a significant increase in thromboembolic events compared with DOACs (RR 6.02; 95% CI 1.02–35.94), a finding largely driven by the results of OCEANIC‐AF, which was terminated early due to excess stroke and systemic embolism with asundexian versus apixaban despite lower bleeding rates.

This was reinforced by meta‐analyses restricted to the atrial fibrillation setting, pooling PACIFIC‐AF, OCEANIC‐AF, and AZALEA‐TIMI 71 results: Emara et al^91^ and Markides et al^92^ reported a 3‐ to 3.4‐fold increase in ischemic stroke with FXI/XIa inhibitors versus DOACs (RR 3.17, 95% CI 2.18–4.62; OR 3.37, 95% CI 2.18–5.19), alongside a 60%–70% relative reduction in major bleeding. The asundexian‐specific analysis by Arif et al^93^ confirmed the absence of efficacy benefit on ischemic stroke (RR 1.64; 95% CI 0.51–5.25).

These pooled analyses should nevertheless be regarded as provisional. They are currently dominated by phase II trials with small numbers of events, by heterogeneous indications, comparators, and background regimens and, in the atrial fibrillation setting, by the OCEANIC‐AF trial whose early termination drives most of the observed effect. None could incorporate the phase III cerebrovascular evidence. Meta‐analyses are therefore likely to become considerably more informative once the pivotal trials are complete: the incorporation of OCEANIC‐STROKE and, in due course, of LIBREXIA‐STROKE and LIBREXIA‐AF will permit indication‐specific pooling with adequate event numbers, allowing the setting dependence of FXI inhibition suggested by the current data to be formally tested rather than inferred.

### Patient Stratification: Where FXIa Inhibition May Fit Best

A question that precedes any discussion of patient selection is whether FXIa inhibitors are intended to be added to antiplatelet therapy, to replace it, or whether this remains undetermined. In secondary prevention after non‐cardioembolic stroke these agents have been evaluated exclusively as an addition to antiplatelet therapy: PACIFIC‐Stroke, AXIOMATIC‐SSP, OCEANIC‐STROKE, and LIBREXIA‐STROKE all administer the study drug on top of single or dual antiplatelet treatment, and none has tested an FXIa inhibitor as a substitute for aspirin or clopidogrel. In atrial fibrillation, by contrast, asundexian was tested as a replacement for a DOAC and proved inferior to apixaban. Current evidence therefore supports an add‐on strategy in non‐cardioembolic stroke and argues against substitution for either antiplatelet therapy or established anticoagulation. It must also be emphasized that this positioning relies on a single positive phase III trial (OCEANIC‐STROKE) without an active comparator: no study has compared FXIa inhibition with intensified or prolonged antiplatelet therapy, and confirmation from LIBREXIA‐STROKE is awaited.

Within the add‐on framework, the positive OCEANIC‐STROKE results, combined with the consistently favorable bleeding profile across phase II and III trials, suggest that the most rational future role for FXIa inhibition may lie in a precision‐medicine strategy guided by clinical‐, imaging‐, and biomarker‐based risk stratification. The ideal candidate may be a patient with meaningful residual ischemic risk despite optimal antiplatelet therapy, yet with subclinical hemorrhagic vulnerability that would make conventional antithrombotic intensification clinically unsustainable.

CMBs represent the prototypical imaging biomarker of this phenotype. The MICON individual‐patient‐data meta‐analysis showed that ≥5 CMBs in patients with recent ischemic stroke or TIA are associated with a markedly higher absolute risk of symptomatic ICH on antithrombotic therapy, with the competing hazard of bleeding approaching or exceeding that of recurrent ischemia in selected subgroups.^6^ Composite small‐vessel disease scores integrating lacunes, white‐matter hyperintensities, perivascular spaces, and CMBs, validated by Staals et al^94^ and refined by Charidimou et al,^95^ also independently predict recurrent stroke and ICH. Finally, a history of hemorrhagic transformation of the index infarct identifies an additional population with disrupted neurovascular integrity in whom antithrombotic intensification is poorly tolerated.^96^, ^97^ The PACIFIC‐Stroke MRI analysis reported that despite CMBs being present in ∼35% of patients and predicting hemorrhagic transformation, asundexian did not accelerate microbleed accumulation (OR 0.96; 95% CI 0.66–1.41).^77^

Conversely, symptomatic large‐artery atherosclerosis, branch atheromatous disease, multiple acute lesions in different vascular territories, and non‐lacunar embolic infarct patterns identify patients in whom an additional anticoagulant mechanism is most likely to yield benefit.^98^ These findings align with the exploratory PACIFIC‐Stroke MRI sub‐study, which reported a numerical reduction in recurrent ischemic events with asundexian 50 mg among patients whose baseline imaging suggested embolic or large‐artery mechanisms (HR 0.71; 95% CI 0.45–1.11), but not among those with isolated lacunar infarcts (HR 1.14; 95% CI 0.62–2.10).^75^

By contrast, patients whose index event is a lacunar infarct attributable to small‐vessel disease are unlikely to be appropriate candidates. In this subtype, the main physiopathologic mechanism is intrinsic arteriolar disease rather than thromboembolism,^99^ the risk of ICH is comparatively high, and antiplatelet therapy (aspirin, clopidogrel, or short‐term dual antiplatelet treatment followed by monotherapy) remains the guideline‐recommended standard.^34^ The absence of benefit with asundexian in patients with isolated lacunar infarcts^75^ is consistent with this reasoning, and current evidence is not sufficient to support FXIa inhibition in this population.

Clinical scores such as CHA_2_DS_2_‐VASc and HAS‐BLED, central in atrial fibrillation, discriminate poorly for intracranial hemorrhage (c‐statistic 0.50–0.65) because most of their predictors, that is, age, hypertension, and prior stroke, simultaneously drive both ischemic and hemorrhagic risk,^100^ while blood biomarkers of vascular inflammation such as IL‐6 and hsCRP independently predict recurrent stroke and may further refine risk stratification.^101^

A further scenario in which the hemostasis‐sparing profile of FXI inhibition may be particularly relevant is breakthrough ischemic stroke, defined as ischemic stroke occurring despite ongoing oral anticoagulation, usually for atrial fibrillation. This is an increasingly recognized high‐risk phenotype for which no therapeutic strategy is established. In the prospective multicenter RENO‐EXTEND study, 1240 patients with atrial fibrillation who sustained an ischemic stroke while receiving a DOAC experienced 207 ischemic or bleeding events over a mean follow‐up of 15 months, corresponding to an annual rate of 13.4%.^102^ The underlying mechanisms are heterogeneous rather than uniformly cardioembolic. In the ASPERA‐R study, which collected breakthrough strokes occurring on continuous anticoagulation across 35 centers, competing non‐cardioembolic etiologies, predominantly large‐artery atherosclerosis, were identified in approximately one quarter of patients and were independently associated with higher 90‐day recurrence, greater disability, and higher mortality^103^; analyses of the same cohort have subsequently shown further heterogeneity of outcome according to sex^104^ and to the presence of active cancer,^105^ while prospective data collection is ongoing (ASPERA‐P, NCT06823466). Consistent with a mechanism that anticoagulation alone cannot address, modifying the antithrombotic regimen has not proven beneficial: in RENO‐EXTEND, event rates did not differ between patients who changed and those who continued the original anticoagulant (OR 1.2; 95% CI 0.8–1.7), whereas concomitant antiplatelet therapy independently predicted bleeding (OR 2.8; 95% CI 1.4–5.5),^102^ and subsequent meta‐analytic and target trial emulation data have confirmed the absence of benefit from anticoagulant switching.^106^

This therapeutic void provides a rationale for evaluating FXIa inhibition in these patients because: (1) circulating activated FXI independently predicts ischemic stroke in anticoagulated patients with atrial fibrillation, indicating that FXI‐mediated thrombin amplification persists despite conventional anticoagulation^107^; (2) the predominance of large‐artery atherosclerosis among competing etiologies parallels the atherosclerotic phenotype in which asundexian proved effective in OCEANIC‐STROKE; (3) the favorable bleeding profile of the class makes antithrombotic intensification conceivable in a population where conventional agents would carry an unsustainable hemorrhagic risk. Given the OCEANIC‐AF result, however, FXIa inhibition could not be expected to replace anticoagulation in atrial fibrillation, and would need to be evaluated as an add‐on strategy in dedicated randomized trials.

All these observations support a multimodal strategy in which FXIa inhibition is targeted at patients whose clinical, imaging, and biomarker profile simultaneously indicates high ischemic risk and intermediate‐to‐high hemorrhagic vulnerability, that is, the phenotype in which the hemostasis‐sparing property of this class is most likely to translate into net clinical benefit.

A question that remains open is whether measurement of coagulation factors, including FXI itself, could inform patient selection, treatment monitoring, or risk assessment. For patient selection, the rationale is biologically plausible. A Mendelian randomization analysis supported a causal effect of genetically determined higher FXI levels on ischemic stroke (OR 2.54; 95% CI 1.68–3.84), with the strongest signal for cardioembolic stroke (OR 4.23; 95% CI 1.94–9.19).^28^ Moreover, higher circulating FXI has been associated with incident ischemic stroke in nested case–control analyses.^108^ More directly relevant to treatment selection, circulating activated FXI, measured with a thrombin‐generation‐based assay, independently predicted ischemic stroke and cardiovascular death in anticoagulated patients with atrial fibrillation,^107^ suggesting that a subset of patients retains an FXI‐dependent thrombotic phenotype despite conventional anticoagulation. No trial, however, has prospectively stratified patients by baseline FXI level or FXIa activity, and none has validated these measurements as predictive biomarkers of treatment response. Moreover, future appropriate assays should be class dependent: FXI clotting activity reflects target suppression for ASO, whereas monoclonal antibodies require antigenic or specific thrombin‐generation methods, and small‐molecule FXIa inhibitors, which do not lower FXI protein levels, would require FXIa‐specific activity or contact pathway‐triggered thrombin‐generation assays that presently remain research tools.^109^

A relevant question for patient stratification is also how FXI inhibitors may relate to platelet function testing, which is already used to assess the response to aspirin and P2Y12 inhibitors. Platelet function assays and CYP2C19 genotyping detect insufficient platelet inhibition,^110^, ^111^ therefore identifying a problem of antiplatelet efficacy for which an evidence‐based solution exists: in the CHANCE‐2 trial, genotype‐guided escalation to ticagrelor in loss‐of‐function carriers reduced recurrent stroke compared with clopidogrel.^111^ In a patient with recurrence or documented high on‐treatment platelet reactivity, the appropriate first step should thus be to optimize antiplatelet therapy, confirming adherence and switching clopidogrel to ticagrelor or another P2Y12 inhibitor rather than to add an anticoagulant. FXIa inhibition addresses a conceptually distinct problem, namely the residual thrombotic risk that persists despite adequate platelet inhibition, acting on thrombin amplification while leaving platelet reactivity unchanged; it would therefore be considered after antiplatelet therapy has been optimized, not as a remedy for antiplatelet non‐response. However, none of the completed stroke trials selected or stratified patients by platelet function, genotype, or any coagulation biomarker: this proposed workflow should therefore be considered a mechanistically rational proposal rather than an evidence‐based algorithm.

## Conclusions

FXI is a compelling target for secondary stroke prevention, grounded in a robust pathophysiological rationale: it amplifies thrombin generation and stabilizes pathological thrombi while contributing marginally to physiological hemostasis. This underpins the concept of hemostasis‐sparing anticoagulation, aimed at reducing thrombotic risk without the bleeding liability of conventional anticoagulants.

OCEANIC‐STROKE provides the first phase III evidence that oral FXIa inhibition reduces recurrent ischemic stroke when added to antiplatelet therapy in non‐cardioembolic stroke, with a 26% relative risk reduction and no significant excess of major or intracranial bleeding, validating the therapeutic hypothesis in patients with atherosclerotic or embolic mechanisms. Conversely, the failure of asundexian in OCEANIC‐AF shows that FXI inhibition alone is insufficient in atrial fibrillation, where tissue‐factor‐driven coagulation predominates and DOACs remain the standard. These divergent outcomes indicate that FXI inhibition is context dependent. In secondary prevention after non‐cardioembolic stroke, where the comparator is placebo on antiplatelet background, low‐intensity FXIa inhibition appears to provide net benefit. LIBREXIA‐STROKE will determine whether milvexian confirms this paradigm.

The absence of a validated reversal strategy remains a substantial barrier to implementation: for long‐acting parenteral agents, the anticoagulant effect cannot be reversed within a clinically useful timeframe, a consideration that currently favors short‐acting oral FXIa inhibitors in the cerebrovascular setting.

Precision‐medicine approaches integrating clinical‐, imaging‐, and biomarker‐based stratification may optimize patient selection. In practical terms, however, the choice between an FXIa inhibitor and currently available options will require individualized clinical judgment rather than a uniform rule. Relevant considerations include: (1) the stroke subtype and presumed vascular mechanism, since benefit has so far been confined to non‐cardioembolic atherosclerotic strokes and is absent in lacunar small‐vessel disease, where antiplatelet therapy remains the standard; (2) the adequacy of current antiplatelet therapy, including adherence and, where available, platelet function or genotype data; (3) the individual hemorrhagic risk profile, in particular the burden of cerebral small‐vessel disease; (4) the maturity of the supporting evidence, at present a single positive phase III trial awaiting confirmation; (5) patient‐specific factors such as renal and hepatic function, co‐treatments with CYP3A4/P‐gp interaction potential, and the feasibility of rapid interruption should bleeding occur. Until confirmatory data are available, FXIa inhibition is best regarded as an adjunctive option for patients who remain at high risk of recurrent ischemic stroke despite guideline‐recommended antiplatelet therapy, rather than as a replacement for it.

## Conflicts of Interest

Dr. Ferrari served as a sub‐investigator in the AXIOMATIC‐SSP, PACIFIC‐STROKE, and LIBREXIA‐STROKE trials. No personal fees or direct compensation were received in relation to this role.

## Funding

The authors declare that no funding was received for the writing of this review.

## Supporting information




Supporting Information

## Data Availability

Data sharing is not applicable to this article as no new data were created or analyzed.
